# Non‐coding RNAs and related molecules associated with form‐deprivation myopia in mice

**DOI:** 10.1111/jcmm.17071

**Published:** 2021-11-28

**Authors:** Shanshan Liu, Huijie Chen, Wenbei Ma, Yanyan Zhong, Yingying Liang, Lishan Gu, Xiaohe Lu, Jiali Li

**Affiliations:** ^1^ Department of Ophthalmology Zhujiang Hospital Southern Medical University Guangzhou China; ^2^ Guangdong Eye Institute Department of Ophthalmology Guangdong Provincial People’s Hospital Guangdong Academy of Medical Sciences The Second School of Clinical Medicine Southern Medical University Guangzhou China

**Keywords:** bioinformatics analysis, myopia, non‐coding RNAs

## Abstract

The role of miRNAs and its regulatory mechanism in myopia are indeterminate. Our study aimed to investigate potential myopia‐associated non‐coding RNAs and related molecules by performing a comprehensive bioinformatic analysis of miRNA expression profile of mice with form‐deprivation myopia (FDM). Differentially expressed miRNAs in two raw microarray data sets (GSE58124 and GSE84220) from Gene Expression Omnibus (GEO) database were comprehensively analysed using GEO2R. Target genes were predicted using miRDB and enriched with Metascape online tool. Protein‐protein interaction (PPI) networks were constructed utilizing STRING and Cytoscape. Significant differentially expressed miRNAs were validated by real‐time polymerase chain reaction (qRT‐PCR) using RNA extracted from monocular FDM ocular tissues. As result, we identified three upregulated miRNAs (mmu‐miR‐1936, mmu‐miR‐338‐5p, and mmu‐miR‐673‐3p) significantly associated with myopia in the two microarray data sets (*p* < 0.05 and |Log (Fold Change) |>1). GO functional analysis suggested these three miRNAs were targeted in genes mostly enriched in morphogenesis and developmental growth of retinal tissues. Enrichment analysis revealed top eight transcription factors, including PAX6 and Smad3, related to myopia. Ten hub genes, including Rbx1, Fbxl3, Fbxo27, Fbxl7, Fbxo4, Cul3, Cul2, Klhl5, Fbxl16 and Klhl42, associated with ubiquitin conjugation were identified. qRT‐PCR confirmed the increased expression of mmu‐miR‐1936 and mmu‐miR‐338‐5p (*p* < 0.05), but no statistical difference was observed in mmu‐miR‐673‐3p expression in myopic retinas. Our findings indicated mmu‐miR‐1936, mmu‐miR‐338‐5p and mmu‐miR‐673‐3p upregulation may be associated with myopia development via post‐transcriptional gene regulation, and identified potential molecules that could be further explored in future studies of the mechanism in myopia.

## 1 INTRODUCTION

Myopia, characterized by a mismatch between eye shape and refractive ability, has become the major cause of worldwide severe vision loss with its rapidly increasing incidence rates.^1^ The worldwide prevalence of myopia is estimated to reach 49.8% by 2050, affecting nearly half the world's population.^2^ Myopia, especially high myopia, can also lead to glaucoma, cataracts, retinal detachment, macular degeneration and other vision‐threatening complications, and even blindness in severe cases.^3^, ^4^ Therefore, it is necessary to clarify the detailed mechanism of myopia to identify potential targets of treatment and ultimately reduce incidence rates.^5^, ^6^, ^7^

Current theories have acknowledged the vital role of scleral remodelling in the development of myopia, initiated through an environmental stimulus, a genetic stimulus or a combination of the two.^8^, ^9^, ^10^, ^11^ During the process of emmetropization, abnormal visual signals in the retina produce unknown molecules that pass to the choroid and then initiate responses in the sclera, leading to an abnormal metabolic process of scleral remodelling, characterized by the decelerated synthesis and accelerated degradation of extracellular matrix (ECM) components.^12^, ^13^, ^14^, ^15^ Several studies indicated that the regulation of gene expression is key to the changes in scleral remodelling caused by visual signals.^16^

microRNAs (miRNAs) are small non‐coding RNA that have assumed a high priority in recent studies due to their ability to modulate gene expression by targeting key epigenetic enzymes.^17^, ^18^ A previous study found that miR‐328 can reduce the expression level of Pax6 by binding to the 3’‐UTR, resulting in an increased expression of MMP2, which can enhance the degradation of extracellular matrix (ECM).^19^, ^20^ As a recognized ECM regulator, miR‐29a has been found to affect the expression of collagen I through Wnt/β‐catenin signalling pathway.^21^ Other miRNAs, such as miR‐184, miR‐29a and miR‐let‐7i, have also been associated with myopia. However, the regulatory mechanism of miRNAs in myopia has not been explicitly described in previous studies.^18^

**FIGURE 1:**
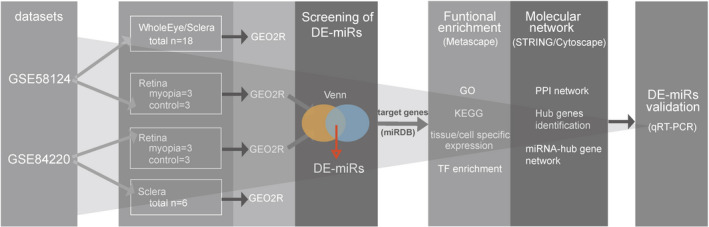
Flow chart of the current study. DE‐miRs, differentially expressed miRNAs; GO, gene ontology; KEGG, Kyoto Encyclopedia of genes and genomes; TF, transcription factors; and PPI, protein‐protein interaction

The purpose of this study was to identify differentially expressed miRNAs involved in the development of form‐deprivation myopia using two microarray data sets (GSE58124 and GSE84220) obtained from Gene Expression Omnibus database (GEO). Deep bioinformatics analysis, including target gene prediction, enrichment analysis, protein‐protein network (PPI) analysis and identification of hub genes, were performed and identified differentially miRNAs were further validated in monocular mouse tissues with form‐deprivation myopia using real‐time polymerase chain reaction (qRT‐PCR). The flow diagram of the analyses is shown in Figure 1.

## 2 MATERIAL AND METHODS

### 2.1 Non‐coding RNA microarray profile collection

Gene Expression Omnibus (GEO) database (http://www.ncbi.nlm.nih.gov/geo) is an online public genome database that stores high‐throughput gene expression data sets.^22^ miRNA expression profiles examined in our study were retrieved from GEO by searching for the following key terms: (‘microRNA’ OR ‘miRNA’) AND ‘form‐deprivation myopia’.

The inclusion criteria for selecting the data sets for assessing the regulation mechanism of retina in myopic sclera were as follows: (i) profiles containing miRNA expression levels of retina samples, (ii) the myopia modelling was form deprivation instead of lens‐induction, and (iii) the organism set was retained, with mouse assuming precedence.

The process resulted in the selection of two gene expression profiles (GSE58124 and GSE84220), separately based on GPL22134 and GPL15880 platforms (GPL15880, Agilent‐029152 Unrestricted Mouse miRNA v15.0 microarray). GSE58124 consisted of six whole eye, three retina and three sclera samples, whereas GSE84220 contained three retina and three sclera samples. Both chips contained an equal amount of normal tissues, with the other eye as a control. All miRNA information was downloaded for subsequent analysis.

### 2.2 Differentially expressed miRNA (DE‐miR) screening

All raw data were then analysed by using GEO2R tools to identify differentially expressed miRNAs between the myopic eye and contralateral control eye. The cut‐off criteria were *p* < 0.05 and |Log (Fold Change) | >1. The GSE58124 and GSE84220 data set were analysed separately, and a heat map was constructed using the limma package in the R Studio (version 1.3.959). Volcano plots were constructed using Prism (version 8.4.0), and overlapping miRNAs from the two data sets were represented on a Venn diagram. The intersecting portion of the Venn diagram was defined as differentially expressed miRNAs, DE‐miRs.

Except for retinas, miRNA expression level data of other ocular tissues (sclera and whole eye samples) were available. All miRNA information was analysed in the two GEO sets using GEO2R; however, only DE‐miRs screened out of the retina samples were selected for subsequent analyses.

### 2.3 Target gene prediction and enrichment analysis

The potential targets of DE‐miRs were predicted using the miRDB (http://www.mirdb.org/) databases.^23^, ^24^ Metascape (http://metascape.org/gp/index.html#/main/step1) is a simple and powerful tool for gene function annotation, analysis and visualization; it integrates many authoritative data resources such as Gene Ontology (GO), Kyoto Encyclopedia of Genes and Genomes (KEGG), UniProt and Drugbank.^25^ In this study, transcription factors, tissue‐/cell‐specific expression, GO and KEGG pathway analyses of target gene prediction were performed via Metascape.

### 2.4 Protein‐protein interaction (PPI) network creation and modular analysis

All predicted target genes of the DE‐miRs were uploaded to STRING (version 11.0) to obtain the PPI pairs and subsequently imported into NetworkAnalyst (https://www.networkanalyst.ca) for visualization.^26^, ^27^, ^28^, ^29^ Organism was limited to ‘Mus musculus’, and a minimum required interaction score >0.400 (medium confidence) was considered significant. Cytoscape (version 3.8.0) is an open source software platform for visualizing complex networks and integrating these with any type of attribute data.^29^ After the PPI relation pairs were obtained, functional modules were figured out by cluster analysis using the Molecular Complex Detection (MCODE) plug‐in of Cytoscape. Analysis parameters were as follows: clusters were found in whole network and ‘haircut’ was selected instead of ‘fluff’, degree cut was 2, node density cut‐off was 0.1, node score cut‐off was 0.2, k‐core was 2, and maximum depth was 100.

### 2.5 Identification of hub genes

After PPI network construction and cluster analysis based on MOCDE, hub genes were selected using another useful plug‐in of Cytoscape, cytoHubba. cytoHubba is used to predict and explore important nodes and subnetworks in a given network based on several topological algorithms.^30^ The constructed PPI network was uploaded to the cytoHubba plug‐in of Cytoscape as a target network. Subsequently, node scores were calculated by all twelve methods, and the top ten genes with the highest degree of connectivity (ranked by MCC) were selected as hub genes of the predicted DE‐miR target genes. A miRNA‐hub gene network was then constructed using Cytoscape.

### 2.6 DE‐miR validation by quantitative reverse transcription‐polymerase chain reaction (qRT‐PCR)

This study was approved by the Animal Care and Ethics Committee of Zhujiang Hospital of Southern Medical University, China (NO. LAEC‐2020–111). Three‐week‐old male wild‐type C57BL/6 mice (n = 9) were obtained and raised in the animal experimental centre of Zhujiang Hospital of Southern Medical University, and the entire experiments were carried out according to the rules and regulations of the centre. Form‐deprivation myopia was induced by the placement of hand‐made caps from 0.2 mL PCR tubes onto a randomly selected eye for 10 days, as described previously.^31^, ^32^ C57BL/6J mice were euthanized according to the approved experimental protocol. Fresh retinal specimens from both treated and control eyes were separated under a stereomicroscope and snap frozen in liquid nitrogen. Total miRNA was isolated from each sample using E.Z.N.A.^TM^ miRNA Kit (R6842‐00, Omega, Bio‐Tek, Norcross, GA, USA) following the manufacturer's protocol. cDNA was synthesized using miRNAFirst Strand cDNA Synthesis (Stem‐loop Method NO. B532453, Sangon Biotech, Shanghai, China). qPCR was performed on a Bio‐Rad real‐time PCR instrument (CFX96 Connect, Bio‐Rad, Hercules, CA, USA) using miRNA qPCR Kit (SYBR Green Method NO. B532461, Sangon Biotech, Shanghai, China). The relative miRNA level was normalized to the internal control U6 using the 2^−∆∆CT^ method. *p* value < 0.05 was considered statistically significant.

## 3 RESULTS

### 3.1 Identification of differentially expressed miRNAs

**FIGURE 2:**
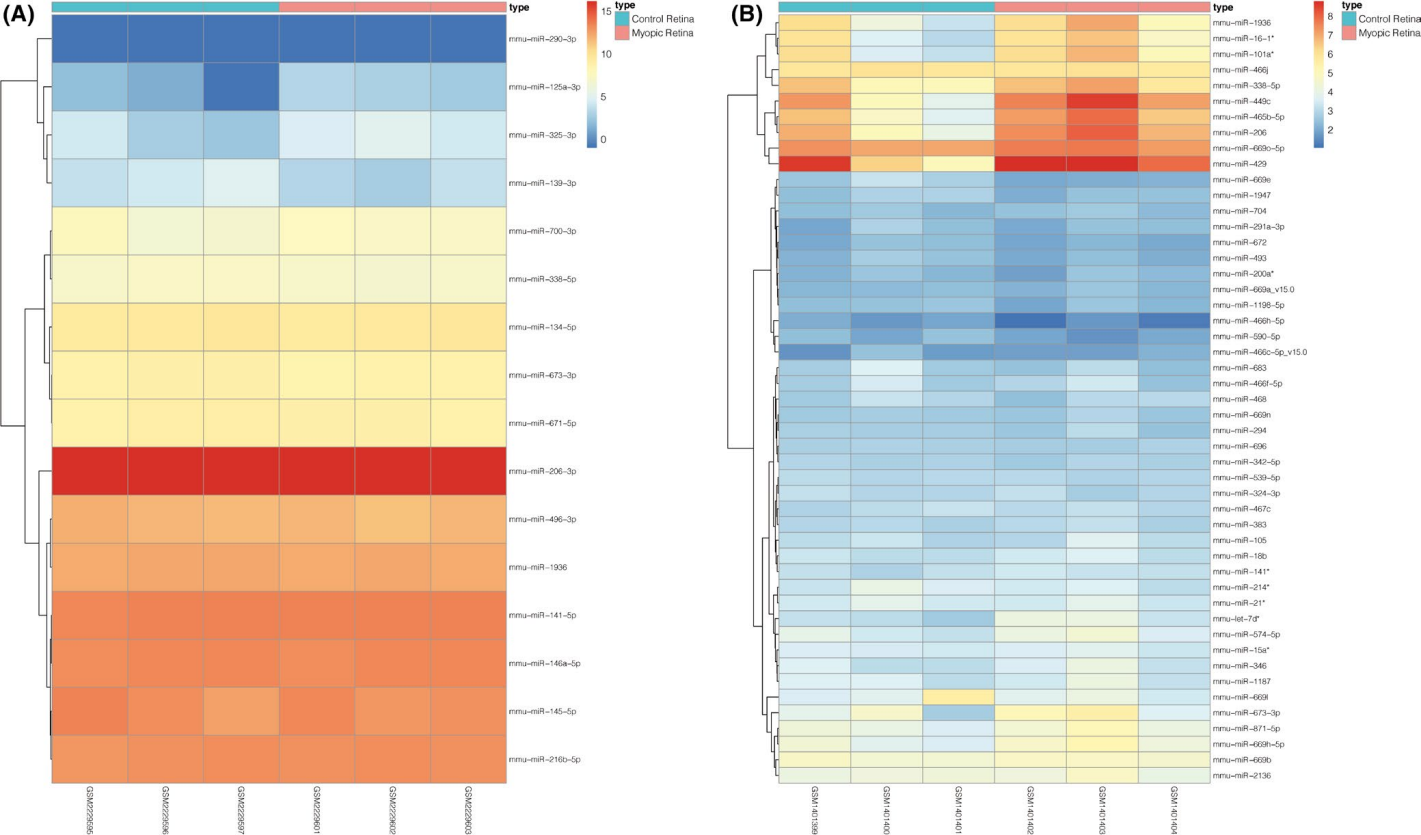
Heat maps of differentially expressed miRNAs between a treated eye (myopia retina) and contralateral untreated eye (control retina) in two microarray profiles. (A) Heat map of GSE84220 data set; (B) heat map for GSE58124 data set (only 49 unique miRNAs are shown of a total of 115 microRNAs)

**FIGURE 3:**
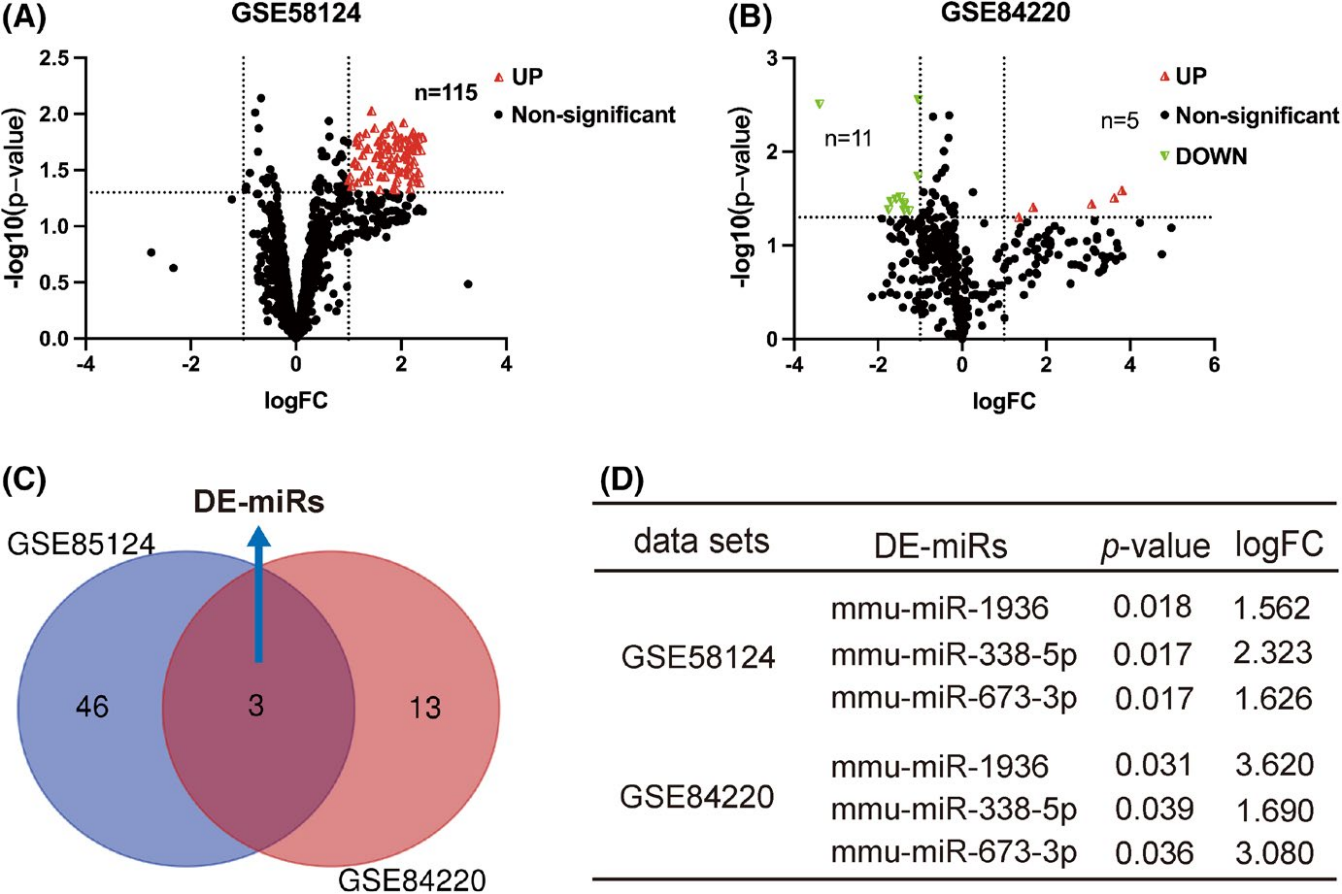
Volcano plots and Venn diagram of this study. (A, B) Volcano plots of the distribution of all differentially expressed miRNAs that were selected with a *p* value < 0.05 and |Log (Fold Change) | >1. Upregulated, downregulated and not significantly changed miRNAs are marked, respectively, as red, green and black dots. From the retina tissues in the data set, a total of 115 miRNAs were differentially expressed in the GSE58124 data sets, and all were upregulated. Sixteen differentially expressed miRNAs (5 upregulated and 11 downregulated) were screened out from the GSE84220 data sets. (C) Venn diagram of co‐expressed miRNAs within GSE58124 and GSE84220, with consensus defined as DE‐miRs. (D) mmu‐miR‐1936, mmu‐miR‐338‐5p and mmu‐miR‐673‐3p were screened out as DE‐miRs; their specific *p* and logFC values are listed in the two data sets

All raw data were uploaded to GEO2R to evaluate the differentially expressed miRNAs using the criterion: *p* value < 0.05 and |Log (Fold Change) | >1. From retina tissues in the data sets, sixteen differential expressed miRNAs (5 upregulated and 11 downregulated) were screened out from the GSE84220 data sets. A total of 115 miRNAs (49 unique miRNAs) were differential expressed in the GSE58124 data sets, and all were upregulated. The differentially expressed unique miRNAs of the two data sets are displayed by separate heat maps in Figure 2. Volcano plots (Figure 3A,B) and a Venn diagram (Figure 3C) were used to highlight the differential expressed miRNAs and the overlap of the two data sets respectively. Overall, we identified three differentially upregulated miRNAs, defined as DE‐miRs: mmu‐miR‐1936, mmu‐miR‐338‐5p and mmu‐miR‐673‐3p (Figure 3D).

In scleral tissues, no differentially expressed miRNAs were found in both data sets. However, in whole eye samples, we identified 39 upregulated miRNAs (Table S1), although the DE‐miRs of these samples were not further analysed.

### 3.2 Target gene prediction and enrichment analysis

**FIGURE 4:**
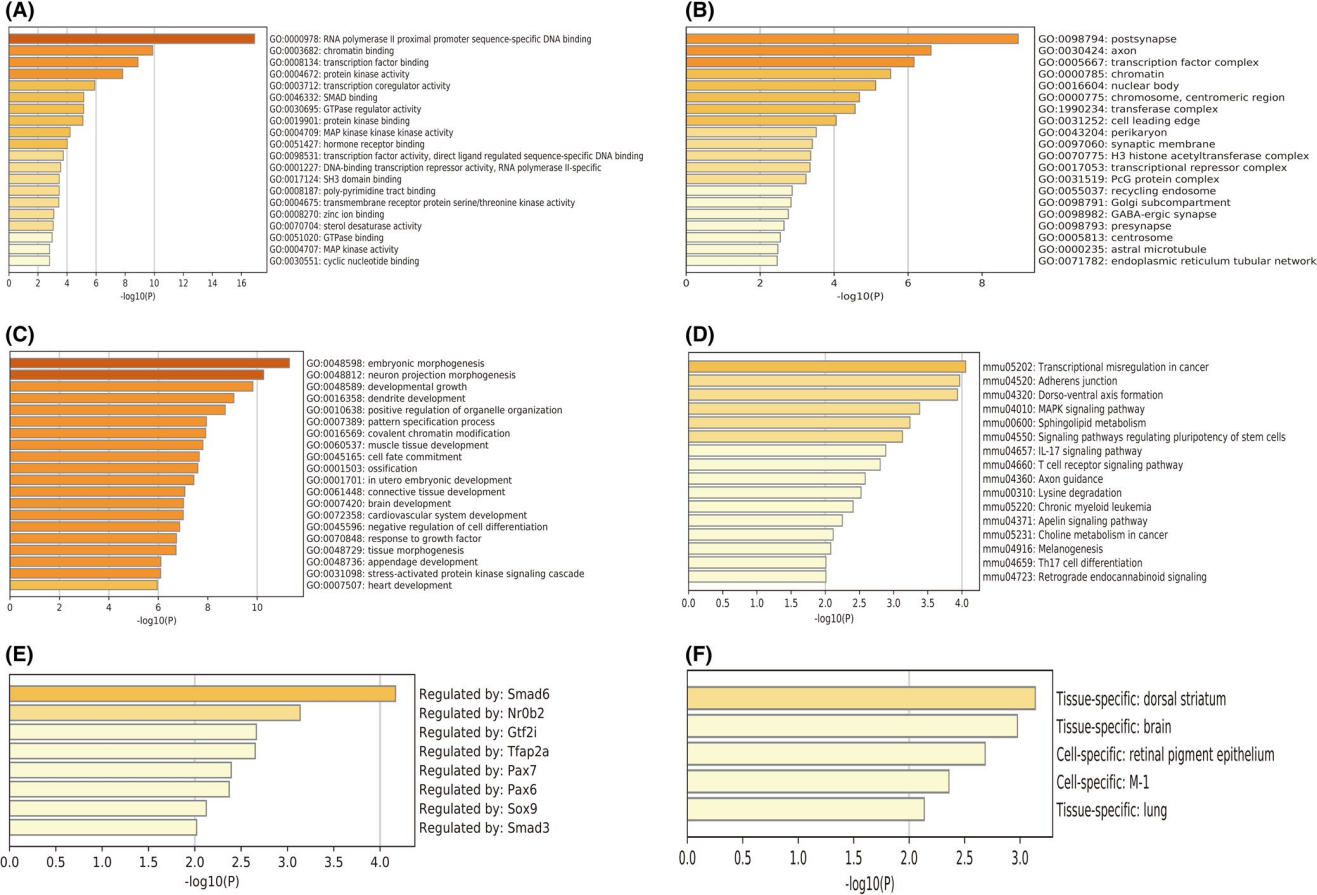
Enrichment analysis of genes predicted from DE‐miRs using Metascape including (A) molecular functions, (B) cellular components, (C) biological processes, (D) KEGG pathways analysis, (E) transcription factor enrichment and (F) tissue/cell expression analysis. DE‐miRs, differentially expressed miRNAs. The length of each bar is equal to −log10(P)

A total of 1340 unique target genes were predicted by an online bioinformatics tool miRDB as presented in Table S2. GO was performed using Metascape and included three categories: molecular function (MF), cellular component (CC) and biological process (BP). The top 20 clusters with representative enriched terms (one per cluster) are shown in Figure 4A–C. Specifically, the MF target genes were mainly involved in RNA polymerase II proximal promoter sequence‐specific DNA binding, chromatin binding and transcription factor binding (Figure 4A). The BP target genes were mainly associated with embryonic morphogenesis, neuron projection morphogenesis and developmental growth (Figure 4B). The CC target genes were mainly involved in postsynapse, axon and transcription factor complex (Figure 4C).

KEGG pathway enrichment analysis identified transcriptional misregulation in cancer, adherens junction, dorso‐ventral axis formation and MAPK signalling pathway as the top pathways (Figure 4D). Metascape was also used assess tissue‐/cell‐specific expression and showed that the target genes were specifically expressed in retinal pigment epithelium (Figure 4F). Transcription factor enrichment showed potentially targeting of Smad6, Nr0b2, Gtf2i, Tfap2a, Pax7, Pax6, Sox9 and Smad3 (Figure 4E).

### 3.3 Protein‐protein interaction (PPI) network analysis and modular analysis

**FIGURE 5:**
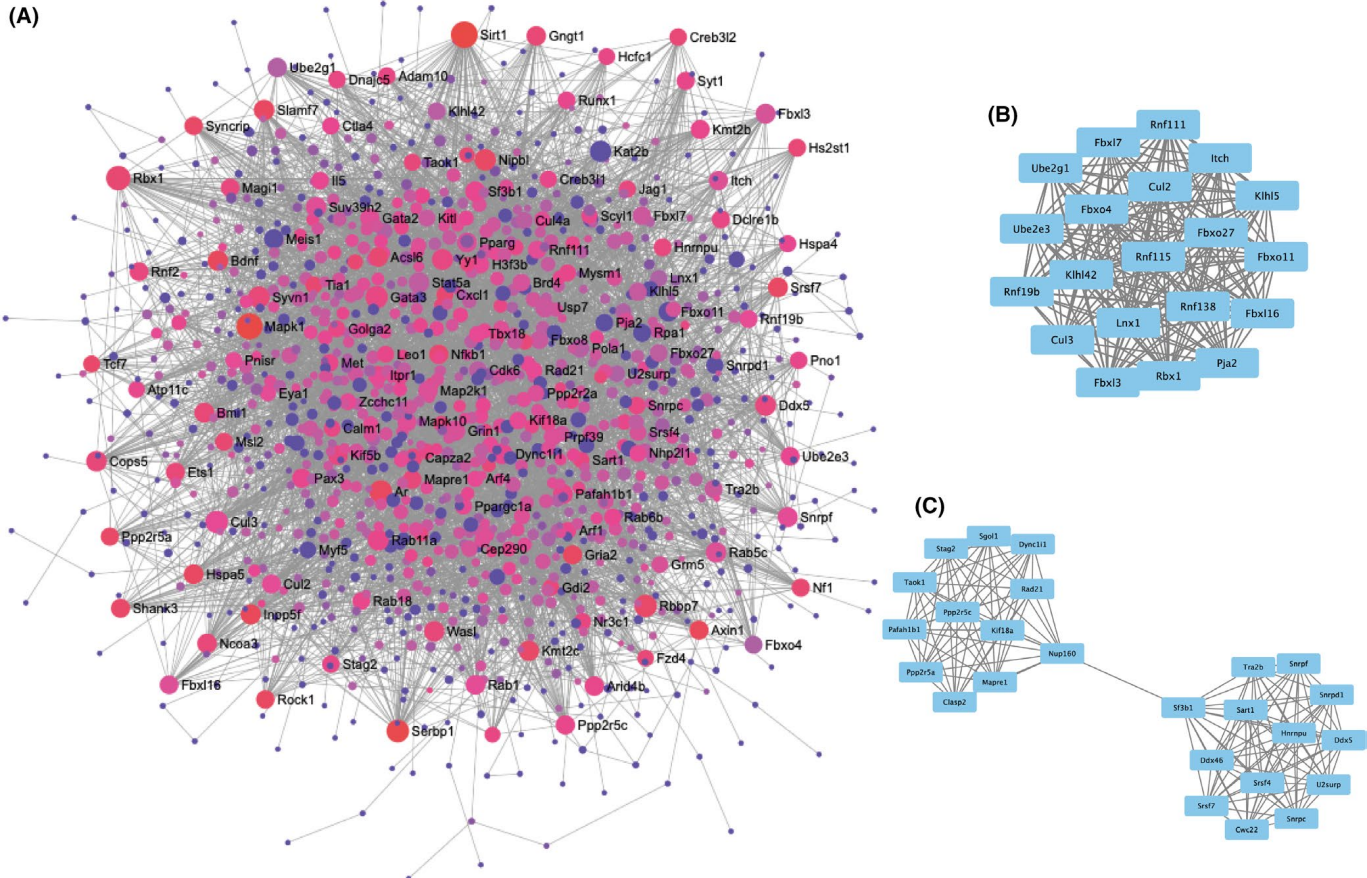
Protein network analysis of 1340 unique target genes. (A) Protein‐protein‐interaction (PPI) network constructed using STRING database and visualized by NetworkAnalyst. The degree of connectivity is shown by the size of the node, with larger circles indicating greater connectivity. The colour of the node represents the value of the betweenness from red (large) to purple (small). (B, C) Top two clusters of functional modules as determined by using the Molecular Complex Detection (MCODE) plug‐in of Cytoscape (version 3.8.0)

The PPI network is the relationship network of biomolecules, which plays an important role in life movement. Identifying the functional modules in a complex interactome can therefore help us to understand the pathogenesis of myopia. STRING database was used to assess functional associations, and a PPI network was constructed to include 1330 nodes and 5644 edges, which was visualized using NetworkAnalyst (Figure 5A). In the protein network graph, each node represents a protein and the edge represents a connection between two proteins. A total of 36 clusters were screened out using the MCODE app, and the top two clusters are presented in Figure 5B–C. Cluster 1 manifests the interaction between 20 nodes, with 190 edges and a score (density×#Nodes) of 20, while in cluster 2, the protein complex includes 25 nodes and 144 edges, with a score (density×#Nodes) of 12.

### 3.4 Hub gene analysis and miRNAs‐hub gene network construction

**FIGURE 6:**
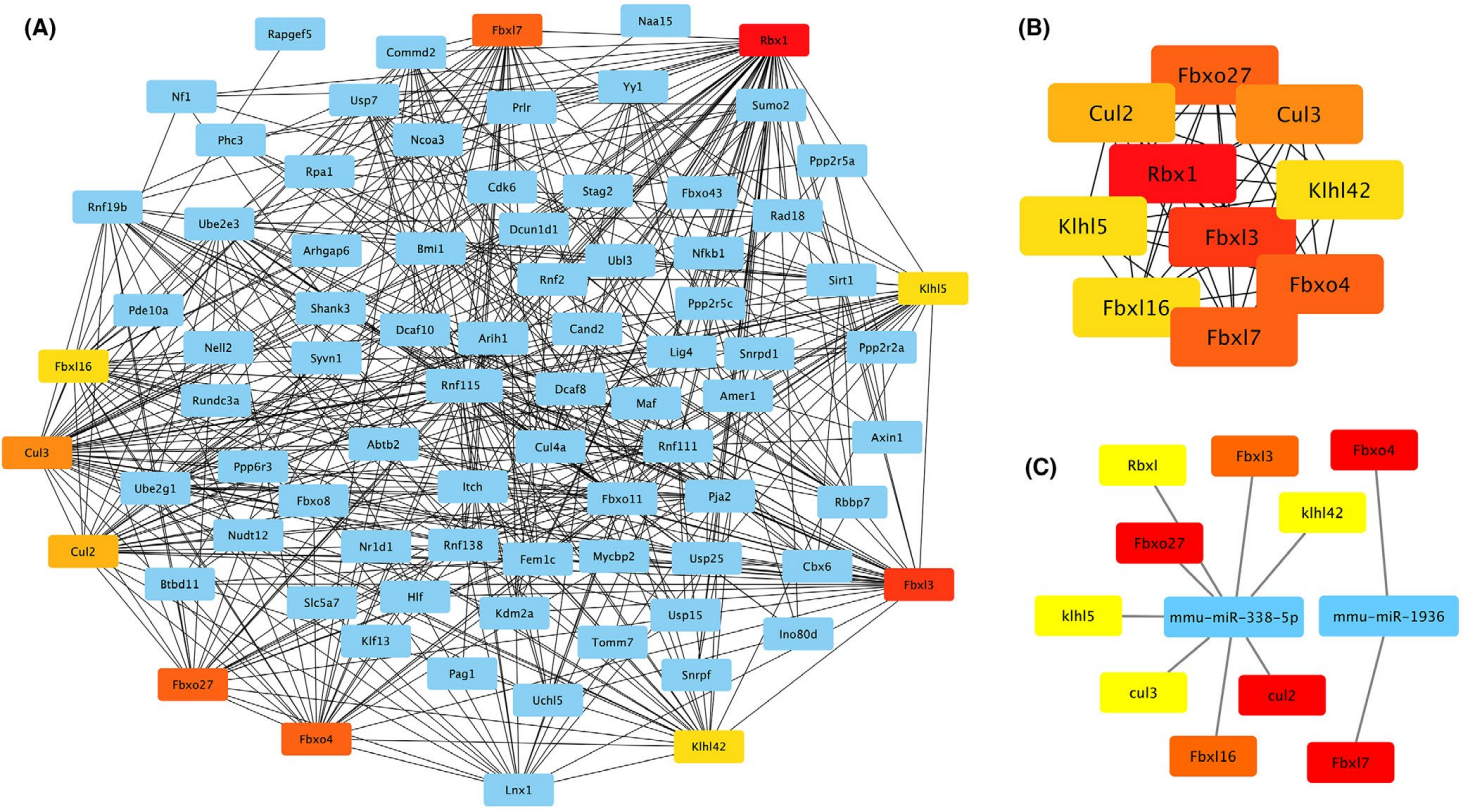
Network of the top 10 hub genes based on cytoHubba plug‐in of Cytoscape. (A) The expanded subnetwork is displayed. The warm colours represent the genes; the blue circles represent genes that directly interact with them. (B) The shortest path is displayed. The darker the node, the higher the ranking using the degree algorithm. (C) Network map of miRNA–hub gene interactions. Rbx1, Fbxl3, Fbxo27, Cul3, Cul2, Klhl42, Klhl5 and Fbxl16 are target genes of mmu‐miR‐338‐5p; Fbxo4 and Fbxl7 are target genes of mmu‐miR‐1936; mmu‐miR‐673‐3p has no target genes that is the core gene in the molecular network

Hub gene analysis identified ten genes that could be regulated by the three differentiated miRNAs (Figure 6A–B): Rbx1, Fbxl3, Fbxo4, Fbxl7, Fbxo27, Cul3, Cul2, Klhl42, Klhl5 and Fbxl16 (ranked according to the degree algorithm). Of all the key nodes, Rbx1 ranked first, suggesting it plays an important role in the formation of form‐deprivation myopia. The network of the ten hub genes with the expanded subnetwork is shown in Figure 6A, and the shortest path is shown in Figure 6B. The integrated result of all the twelve methods used to calculate scores in the cytoHubba plug‐in of Cytoscape is shown in Table S3. The miRNA‐hub gene network is displayed in Figure 6C. mmu‐miR‐338‐5p potentially could target 8 (Rbx1, Fbxl3, Fbxo27, Cul3, Cul2, Klhl42, Klhl5 and Fbxl16) of the10 hub genes, whereas Fbxo4 and Fbxl7 were identified as potential target of mmu‐miR‐1936.

Interestingly, we found that the 10 hub genes were all related to ubiquitination. IUUCD (version 2.0), a family‐based database of ubiquitin and ubiquitin‐like conjugation, was used to obtain the latest information of functional roles of the 10 genes.^33^ The placings, score, gene name, protein name and functional description are shown in Table S4.

### 3.5 Upregulation of mmu‐miR‐1936 and mmu‐miR‐338‐5p in retinal tissues of mice with form‐deprivation myopia

**FIGURE 7:**
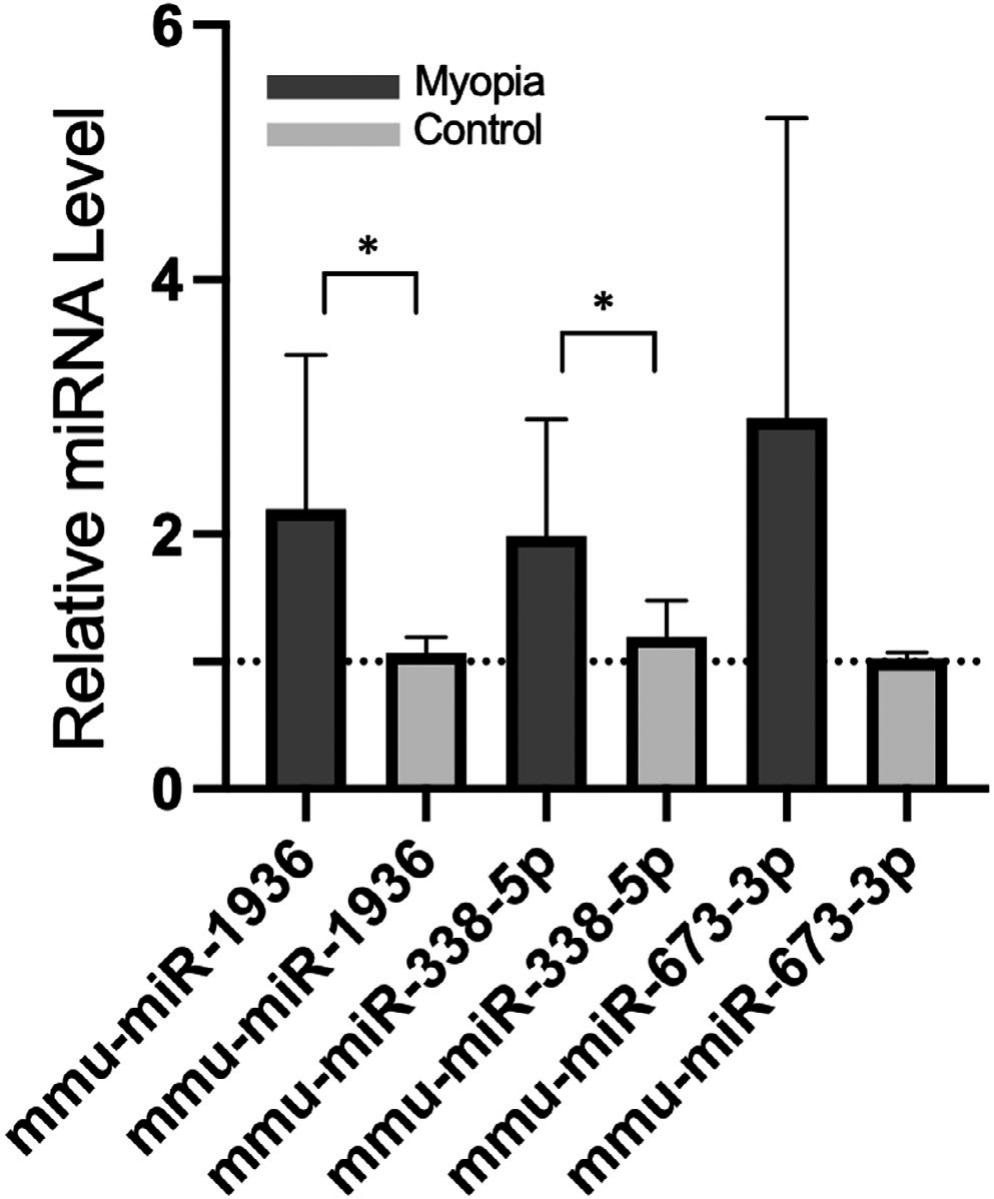
Validation of the relative expressed level of mmu‐miR‐1936, mmu‐miR‐338‐5*p* and mmu‐miR‐673‐3*p*. *p < 0.05. The fold changes were normalized to small nuclear RNA U6 and calculated using the 2^−∆∆CT^ method. The validation results show that the expression levels from myopia of mmu‐miR‐1936 and mmu‐miR‐338‐5p in retinal tissues were statistically significantly higher than those of controls

We further verified the expression level of miRNAs using qRT‐PCR analysis. As shown in Figure 7, the expression levels of mmu‐miR‐1936 and mmu‐miR‐338‐5p in retinal tissues of myopic eyes were statistically significantly higher than those of controls, but there was no statistical difference in mmu‐miR‐673‐3p levels.

## 4 DISCUSSION

In present study, we identified three upregulated miRNAs (mmu‐miR‐1936, mmu‐miR‐338‐5p and mmu‐miR‐673‐3p) in the retina of mice with form‐deprivation myopia based on the results of overlapping two microarray data sets (GSE58124 and GSE84220) from the Gene Expression Omnibus (GEO) database. We verified the abnormal expression of two of the three miRNAs using qRT‐PCR and subsequently performed a comprehensive bioinformatic analysis, providing a framework for understanding the molecular mechanisms underlying the role of these miRNAs in form‐deprivation myopia.

In our study, we found three differential expressed miRNAs (mmu‐miR‐1936, mmu‐miR‐338‐5p and mmu‐miR‐673‐3p) associated with myopia. Currently, there are few studies on these three miRNAs, most of which have focused on cancer.^34^, ^35^, ^36^ Previous studies found that miR‐338‐5p promoted glioma cell invasion by regulating matrix metalloproteinases 2 (MMP2), which has been implicated in the development of myopia.^37^, ^38^ Given the fact that miRNAs can mediate ECM remodelling via MMP2, a process that drives tumour invasion, it may be possible that these miRNAs influence myopic scleral ECM remodelling in a similar way during visual manipulation in form‐deprivation myopia. We surmise that the retina is involved in the regulation of myopic sclera through DE‐miRs; however, this process needs to be confirmed by further research.

miRNAs may not directly regulate genetic changes in myopic scleral tissue, but may rather cause changes through the retinal pigment epithelial (RPE) layer. Our enrichment analysis of tissue‐ and cell‐specific expression showed that target genes of DE‐miRs were mainly expressed in RPE cells. Functional enrichment analysis indicated that the DE‐miRs were involved in embryonic morphogenesis, developmental growth and pattern specification processes, which fit well with the nature of myopia: an abnormal development of the eyeball. The eye is a typical system in the field of developmental biology.^39^, ^40^ We suspected that the RPE can influence the transmission of chemical signalling from the retina to the sclera and thereby affecting the eye growth.

Lastly, we focused on two specific types of molecular changes that may be associated with myopia: alterations in transcription factors (TF) and ubiquitination related genes. On the one hand, the results of Metascape revealed a potential role of TF in the development of myopia with the enrichment of Smad6, NrOb2, Gtf2i, Tfap2a, Pax7, Pax6, Sox9 and Smad3. Pax6 is known to be an important gene for eye development, and research has related miR‐328 to myopia by its binding to the PAX6 locus.^20^ The elucidation of the whole regulatory network of miRNA‐TF‐mRNA at transcriptional level is helpful for clarifying the changes in the retinal‐sclera axis during myopia.^41^ On the other hand, we used the cytoHubba plug‐in of Cytoscape to screen out the top ten hub genes of the PPI network with the highest connectivity degrees, unveiling Rbx1, Fbxl3, Fbxo4, Fbxl7, Fbxo27, Cul3, Cul2, Klhl42, Klhl5 and Fbxl16, which are all, remarkably, related to ubiquitin conjugation. Ubiquitination is a post‐translational modifications of proteins that plays an important role in regulating the structure and function of proteins.^42^ The current study found the core genes of PPI network constructed by target genes of DE‐miRs to be related to ubiquitination; thus, further investigation of the specific proteins involved in ubiquitination and the interaction of these proteins may open up new perspectives in myopia research and drug targets.^43^, ^44^, ^45^

There are a few limitations in the current study. First, to determine actual differentially expressed miRNAs in myopic retina, a larger sample size would be needed for external verification. Moreover, changes in myopic retina miRNAs/target mRNAs were analysed in the present study. However, there was no direct evidence that the changes in biochemical substances or gene expression in the retina led to the change in RPE layer miRNAs, with consequent action on the sclera to cause myopia. It is worth mentioning that miRNAs can be packaged in structures called extracellular vesicles. These vesicles, including exosomes and microcapsules, are cell‐derived membranous structures that are transferred between cells to establish intercellular communication.^46^, ^47^ Future studies can further clarify the mechanism of visual manipulation‐induced myopia by labelling and tracing retinal exosomes that contain miRNAs.

In summary, this study identified key miRNA signalling factors and their interactions of myopia, expanding our current knowledge of the patterning mechanisms active throughout myopia formation and bringing new insights into the molecular mechanisms underlying form‐deprivation myopia.

## CONFLICT OF INTEREST

The authors declare no competing financial interests.

## AUTHOR CONTRIBUTIONS

**Liu Shanshan:** Conceptualization (lead); Data curation (equal); Formal analysis (equal); Resources (equal); Writing—original draft (equal). **Chen Huijie:** Data curation (equal); Formal analysis (equal); Resources (equal); Writing—original draft (equal). **Ma Wenbei:** Resources (equal); Supervision (equal); Validation (equal); Visualization (equal). **Zhong Yanyan:** Investigation (equal); Project administration (equal); Validation (equal); Visualization (equal). **Liang Yingying:** Writing—original draft (equal); Writing—review & editing (equal). **Gu Lishan:** Data curation (equal); Methodology (equal); Validation. **Lu Xiaohe:** Project administration (equal); Supervision; Writing—review & editing (equal). **Li Jiali:** Conceptualization (equal); Funding acquisition (lead); Supervision; Writing—review & editing (lead).

## Supporting information

Table S1

Table S2

Table S3

Table S4
